# Is the intraosseous access route fast and efficacious compared to conventional central venous catheterization in adult patients under resuscitation in the emergency department? A prospective observational pilot study

**DOI:** 10.1186/1754-9493-3-24

**Published:** 2009-10-08

**Authors:** Bernd A Leidel, Chlodwig Kirchhoff, Viktoria Bogner, Julia Stegmaier, Wolf Mutschler, Karl-Georg Kanz, Volker Braunstein

**Affiliations:** 1 Department of Emergency Medicine, Charité - University Medicine Berlin, Campus Benjamin Franklin, Hindenburgdamm 30, 12203 Berlin, Germany; 2 Department of Trauma, University Medical Centre of Munich, Downtown, Nussbaum Street 20, 80336 Munich, Germany; 3 Helicopter Emergency Medical Service *Christoph 31*, ADAC Luftrettung air rescue services, Charité - University Medicine Berlin, Campus Benjamin Franklin, Hindenburgdamm 30, 12203 Berlin, Germany

## Abstract

**Background:**

For patients' safety reasons, current American Heart Association and European Resuscitation Council guidelines recommend intraosseous (IO) vascular access as an alternative in cases of emergency, if prompt venous catheterization is impossible. The purpose of this study was to compare the IO access as a bridging procedure versus central venous catheterization (CVC) for in-hospital adult emergency patients under resuscitation with impossible peripheral intravenous (IV) access. We hypothesised, that CVC is faster and more efficacious compared to IO access.

**Methods:**

A prospective observational study comparing success rates and procedure times of IO access (EZ-IO, Vidacare Corporation) versus CVC in adult (≥18 years of age) patients under trauma and medical resuscitation admitted to our emergency department with impossible peripheral IV catheterization was conducted. Procedure time was defined from preparation and insertion of vascular access type until first drug or infusion solution administration. Success rate on first attempt and procedure time for each access route was evaluated and statistically tested.

**Results:**

Ten consecutive adult patients under resuscitation, each receiving IO access and CVC, were analyzed. IO access was performed with 10 tibial or humeral insertions, CVC in 10 internal jugular or subclavian veins. The success rate on first attempt was 90% for IO insertion versus 60% for CVC. Mean procedure time was significantly lower for IO cannulation (2.3 min ± 0.8) compared to CVC (9.9 min ± 3.7) (p < 0.001). As for complications, failure of IO access was observed in one patient, while two or more attempts of CVC were necessary in four patients. No other relevant complications, like infection, bleeding or pneumothorax were observed.

**Conclusion:**

Preliminary data demonstrate that IO access is a reliable bridging method to gain vascular access for in-hospital adult emergency patients under trauma or medical resuscitation with impossible peripheral IV access. Furthermore, IO cannulation requires significantly less time to enable administration of drugs or infusion solutions compared to CVC. Because CVC was slower and less efficacious, IO access may improve the safety of adult patients under resuscitation in the emergency department.

## Background

Success rate and time need for vascular access is crucial in the emergency patient under resuscitation. However, peripheral intravenous (IV) access might be difficult especially in the dehydrated or hemodynamic unstable, injured or critically ill patient with collapsed peripheral veins. Also chemotherapy and long-term IV drug addiction can lead to inaccessible peripheral veins. Failure rates of IV access in emergency situations are described between 10-40% [1-3]. Reattempts to gain vascular access lead to valuable time loss with potential subsequent influence of patients' safety. The average time necessary for peripheral IV catheterization is reported to add up to 2.5-13 min, and sometimes even up to 30 min in patients with difficult to access peripheral veins [1-5]. This can lead to a delay of necessary treatment and longer on-scene times [6]. Longer on-scene times might be followed by additional delay in the emergency department, when reattempting vascular access. Also isolated prolongation of on-scene time delays definitive treatment. Time lag of necessary diagnostic and treatment procedures consecutively compromises the emergency patient [7,8]. Therefore, current evidence based practice management guidelines for the prehospital fluid resuscitation in injured patients of the Eastern Association for the Surgery of Trauma (EAST) recommends not to perform vascular access on scene, if it delays patient transport to definitive care [9].

Alternative ways of drug and fluid administration are sublingual, endotracheal, subcutaneous and intramuscular application. However, these options do not really reflect a reasonable possibility, due to its uncertain and uncontrolled administration of substance dosages with incalculable pharmacodynamic and pharmacokinetic effects. Furthermore, only small amounts of certain applicable drugs can be given and volume resuscitation or transfusion of blood products are impossible at all [10,11].

If vascular access is necessary in the acute setting of unstable patients admitted to the emergency department, and peripheral IV cannulation is impossible, central venous catheterization (CVC) is a common alternative procedure [10,11]. Besides providing vascular access for fluid resuscitation, CVC also allows hemodynamic monitoring [12-15]. However CVC is relatively time-consuming and associated with relevant risks for the patient, especially in the emergency setting. Most frequent complications include venous thrombosis, catheter related infections, arterial puncture and pneumothorax [12,14-17].

Consequently, a different vascular access technique may be reasonable to increase patients' safety, at least as a bridging procedure during ongoing resuscitation efforts until the patient is in a more stable condition. In this respect, intraosseous (IO) vascular access is an option, already established in paediatric patients for decades [18,19]. Its importance in adults is less propagated, especially for in-hospital use. In general, IO cannulation of the non-collapsible and highly vasculated intramedullary venousplexus of the concellous bone marrow can provide a rapid, safe and easy vascular access to administer drugs, volume and blood products to the emergency patient. All metaphyseal segments of long bones are filled with highly perfused bone marrow, which are able to transport applied drugs and fluids to the central vascular system rapidly. Transport times are reported to be 1-2 min, even during cardiopulmonary resuscitation with chest compressions [10,11]. Therefore the aim of the present study was to compare the IO access versus CVC regarding success rate of the procedure on first attempt and procedure time needed in adult patients under trauma or medical resuscitation admitted to our emergency department with impossible peripheral IV access. We hypothesised, that CVC is faster and more efficacious compared to IO access.

## Methods

### Study design

This prospective observational study was conducted in the emergency department of an urban level I trauma centre. Consecutive adult injured or critically ill patients (≥18 years of age) brought to our resuscitation bay were included, without or insufficient peripheral IV catheterization and necessary immediate vascular access. A senior attending physician, consultant in surgery and emergency medicine, directed resuscitative efforts following the Advanced Trauma Life Support protocol for injured and the Advanced Cardiac Life Support protocol for ill patients. The local ethics committee approved this study. Written informed consent was obtained from each patient, when returning to full consciousness or from the next of kin or a legal representative. With the present analysis, we evaluated the retrieved data of the first 10 patients included in our study.

### Patient demographics

The patient's baseline characteristics such as age, gender, injury or cause of vital organ disorder were retrieved subsequently if not available on admission. All treatment data and measured parameters assessed in the resuscitation room were prospectively collected and recorded in a structured form for each patient.

### Treatment protocol

During the initial resuscitation in accordance with the present standards of care, peripheral IV access was attempted three times for a maximum of 2 min. If unsuccessful, IO access and CVC was performed simultaneously in a standardized course of action by two independent participants. A third independent observer with two stopwatches took the time of each procedure. The measured time of each procedure was defined as the duration of picking up the prepared set of IO or CVC access device from the shelf, preparation of the access set and patients' insertion site including desinfection and draping, insertion procedure of IO access or CVC itself, assembling of the access set and first successful administration of drugs or infusion solutions through the newly established vascular access. Success rate of the procedure on first attempt was defined as successful administration of drugs or infusion solutions via the performed vascular IO or CVC access on first effort. Failure was defined as more than one (the first) effort to enable drug or infusion solution administration, e.g. due to impossible insertion or advancing the guidewire in CVC. On the other hand, more than one effort to puncture a central vein was not distinct as unsuccessful procedure on first attempt.

### Participants

Each two independent participants were trained consultants and well experienced in resuscitation. Anaesthesiologists performed CVC while surgeons provided the IO access. Each surgical participant received a 60 min lecture on the use and technique of the IO device, including standardized educational videos, a demonstration and an independent, self-performed insertion on the IO model.

### Central venous catheterization

CVC was performed in a standardised procedure using landmark orientated standard Seldinger technique [16,20]. For hemodynamic monitoring option, internal jugular or subclavian vein was preferred to femoral access. According to our protocol, insertion site was primarily subclavian vein for CVC, but a different insertion site was chosen appropriate to injury pattern. For CVC a standard triple- or quad-lumen 7-French catheter (Arrow International Inc., 155 South Limerick Road, Limerick, PA 19468-1699, USA) was used, depending on patients' need. A chest radiograph was obtained in each patient following CVC to confirm placement and assess for complications.

### Intraosseous vascular access

**Figure 1:**
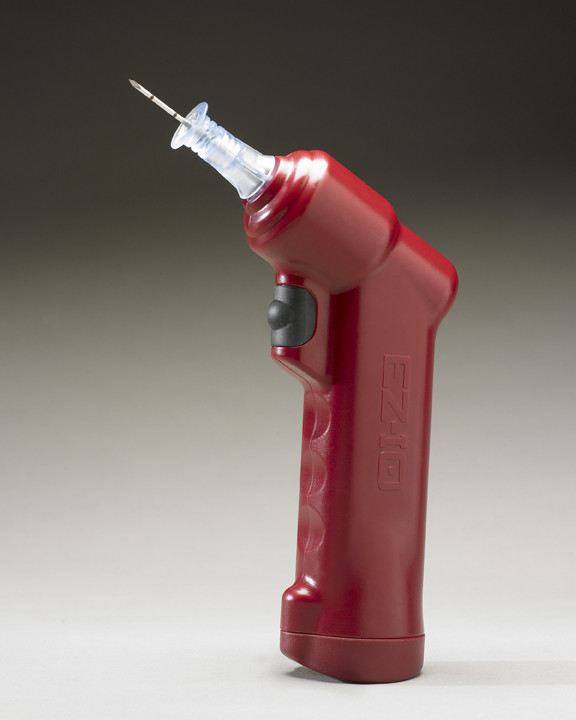
**Battery driven intraosseous vascular access device (EZ-IO^®^, Vidacare^®^)**.

IO access was achieved in a standardised procedure applying landmark orientated standard technique [21-27]. According to our protocol, insertion site was primarily proximal humerus for IO cannulation, but a different insertion site was chosen appropriate to injury pattern. IO access was performed with a battery driven device, the EZ-IO system from Vidacare Corporation (Vidacare Corporation, 722 Isom Road, San Antonio, TX 78216, USA). It contained a lithium battery-powered drill driven handle and a 15-gauge (1.8 mm), 25 mm in length, stainless EZ-IO AD cannula for adults with extension tubing. The reusable 14 × 9 × 5 cm sized driver weighs 455 grams and should provide 1000 insertions or 10 years of shelf life according to the manufacturer (Fig. 1).

**Figure 2:**
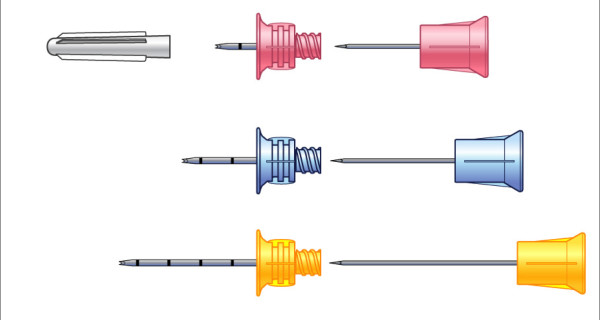
**Different EZ-IO^® ^cannula sets (Vidacare^®^) depending on patients age and excessive tissue over the insertion site** (pink: EZ-IO PD for pediatric patients; blue: EZ-IO AD for adult patients; yellow: EZ-IO LD for obese patients or patients with excessive tissue over the insertion site).

There are three different cannula sizes available depending on patients' age: EZ-IO PD 15 mm in length for paediatric patients from 3-39 kg weight, EZ-IO AD 25 mm in length for adult patients >39 kg weight and EZ-IO LD 45 mm in length for obese patients or patients with excessive tissue over the insertion site (Fig. 2).

Once the IO cannula is attached to the driver, the needle is inserted power-driven under gentle manual pressure. After insertion, the driver and stylet of the cannula has to be removed leaving the IO catheter in place. The inserted catheter has to be attached to the extension tubing and followed by a syringe saline flush before drug and infusion solution administration. FDA approved the EZ-IO for humeral head, proximal and distal tibial (medial malleolus) access in adults, as well as for proximal tibial access in paediatric patients. Contraindications of IO access are rare and relative in cases of life threatening injuries or illness. However, the IO cannula should not be inserted into a fractured bone to avoid extravasation, in the absence of adequate anatomical landmarks due to excessive tissue, if infection at the area of insertion is obvious, and following previous IO access or significant orthopaedic procedure like limb or joint prosthesis at the insertion site. Each IO cannula was removed within 24 hours of insertion according to manufacturers recommendations.

### Follow up and complications

Patient follow up was performed until hospital discharge. If patients were discharged from the hospital within 14 days of admission, a standardized telephone interview with the relevant patients 2 weeks after hospital admission was conducted. Possible complications were determined a priori and a list was defined based on the well-reported complications in literature [16,20-27]. Based on this list, complications occurring under or following vascular access procedures were recorded for each access attempt in all patients with a standardized protocol. This protocol defined the assessment of complications for each patient in the exact same way regarding vascular access type, time and kind of complication. Possible complications included failure of vascular access, malposition, dislodgment, bleeding, compartment syndrome, arterial puncture, haematothorax, pneumothorax, venous thrombosis and vascular access related infection. For instance to assess for complications following CVC, each patient obtained a chest radiograph. To determine vascular access related infection, insertion sites were inspected and documented three times daily. Additionally, every IO cannula and central venous catheter tip was microbiologically examined after its removal.

### Statistical analysis

For statistical testing SPSS version 13.0 software (SPSS, Chigaco IL, USA) was employed. According to the distribution and sample size, for statistical evaluation the Mann-Whitney Rank Sum test was applied to analyze the differences in procedure times between the two vascular access techniques at a significance level of p = 0.05.

## Results

Four women and six men, ranging in age from 18 to 70 (on average 39.7 ± 18.2) years were included. The IO insertion site was proximal tibial in four patients, and humeral in six patients. CVC was achieved in three internal jugular veins, and in seven subclavian veins.

The success rate on first attempt was 90% for IO access versus 60% for CVC. One IO cannulation failed due to operator mishandling by not selecting the correct insertion site at the proximal humerus. The IO catheter did not reach the bone marrow because of the overlying soft tissue at the incorrect insertion site. Four CVC procedures failed at first attempt, requiring at least one more attempt. In all unsuccessful CVC attempts the guidewire was unable to be inserted or advanced into the vessel.

**Figure 3:**
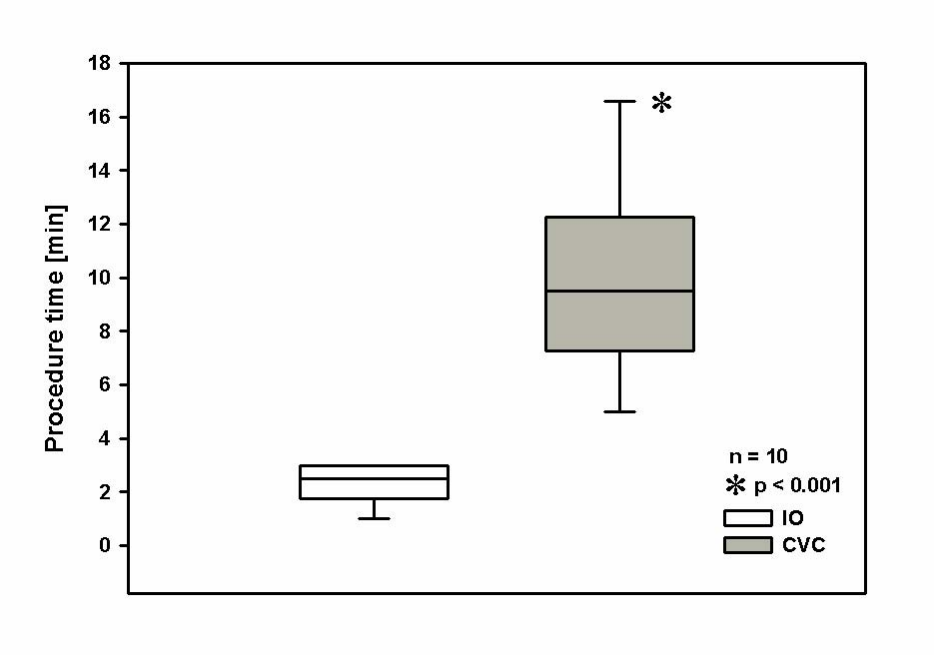
**Procedure time of intraosseous (IO) cannulation was significantly shorter than central venous catheterization (CVC) for vascular access to enable drug and fluid administration in adult emergency patients under resuscitation**.

The mean time required for the vascular access procedure was significantly shorter (p < 0.001) for IO cannulation (2.3 min ± 0.8; time range 1-3 min) compared to CVC (9.9 min ± 3.7; time range 5-17 min) (Fig. 3).

Beside the above mentioned unsuccessful access procedures on first attempt following IO cannulation or CVC, no further complications were detected. Especially no malposition, dislodgment, bleeding, compartment syndrome, arterial puncture, haematothorax, pneumothorax, venous thrombosis and vascular access related infection was observed.

## Discussion

Present guidelines of the Eastern Association for the Surgery of Trauma (EAST) and the National Association of EMS Physicians (NAEMSP) advocate IO access in the prehospital setting, if peripheral venous catheterization is impossible [9,28]. Because lacking peripheral IV access is also an issue in adults under in-hospital resuscitation, we were interested whether IO approach could be an alternative in the emergency department. To our knowledge, no other study has prospectively compared IO access versus standard alternative vascular access procedures in a real scenario for in-hospital patient care yet. Therefore, we investigated the in-hospital IO approach versus CVC in adult patients under resuscitation lacking peripheral IV access. IO cannulation was performed as a bridging procedure and was compared to CVC regarding success rate on first attempt and procedure duration. Our results show that IO vascular access was significantly more successful and required significantly less time when compared to CVC.

Today, there are different techniques of IO access available, also for the adult. Several out-of-hospital trials documented, that IO approach is consistent, rapid, and safe for drug delivery and fluid resuscitation in adults. Different investigators reported successful vascular access within 1-2 min in 72-100% of patients [21-24,26,27]. In two prospective out-of-hospital trials including more than 500 adults, IO cannulation was even successful within 20 sec in 95-97% of patients [21,26].

Only a small number of trials evaluated the isolated utilization of in-hospital IO access in severely injured or critically ill adults. *Ong et al. *published their experience of 24 tibial and 11 humeral successful IO insertions in altogether 24 adults. Success rate at first attempt was 97% (34/35); a second attempt was necessary for one tibial access. All insertions were achieved within 20 sec, relevant complications did not occur [25]. *Valdes *already reported in 1977 about IO access in 15 critically ill adults (18-86 years of age) with a success rate of 87% (13/15). In these patients, an average of 4 litres were administrated for an average of 5.4 days. Complications like infection or emboli did not occur. Application times for IO access were not available [29]. *Iserson *described the successful application of IO access in 22 adults (36-84 years of age) suffering cardiac arrest. Application time was <1 min and complications were not reported [30]. *Cooper et al. *published their experience of 22 successful inserted IO needles in adults in the military combat environment. Success rate was 97%, relevant complications did not occur, especially no infection [31]. In line with these findings, our results show also a high success rate of 90% and a low mean procedure time of 2.3 min. However, our recorded procedure time included not only the IO insertion itself, but also the preparation of the insertion site, the device and its assembling until the administration of drugs or fluids.

The majority of insertion sites for IO access in the adult include the sternum, medial clavicle, proximal humerus, distal radius, proximal tibia, distal tibia and distal fibula [21-27,29-31].

Following an instruction course of a maximum of 2 hours, the safe and rapid use of diverse IO access devices are demonstrated by different studies. Regarding learnability and handling, data reveal success rates of 93-100% in IO access performance within 2 min [32-37].

Most drugs can be administered IO in equivalent dosage and with the same time effect compared to IV. Pharmacodynamic and pharmacokinetic effects of IO applied drugs and infusions are well described in the literature [38-41]. Currently no resuscitation drugs are contraindicated for IO administration. In our study, we mainly administered common resuscitation drugs, beside crystalloid and colloid solutions, red packed cells, and fresh frozen plasma.

Over all, flow rates of IO vascular access are lower than large bore peripheral IV catheters, and depend on patients' age, site of insertion and cannula size. Most IO cannulas for adults are 15-gauge needles, and enable flow rates comparable to a 20-gauge peripheral IV catheter. IO Flow rates on gravidity account between 10-34 ml/min and can be increased up to 80-165 ml/min using a pressure bag [25,42-45]. Therefore IO rapid volume resuscitation is limited, however two or more IO cannulations in the same patient may facilitate fluid therapy [25].

Limitations and risks of IO access have to be considered. According to manufacturers' recommendations, fractured bones should not be cannulated due to the risk of extravasation at the fracture site. Furthermore, to minimize infection risks, areas of limb or joint prosthesis should not be selected as insertion site. IO needles should be removed within 24 hours after insertion and no further IO cannulation should be performed at the same site within 24 hours. Obvious infection at the insertion site contraindicates IO access. However, in life threatening conditions, these contraindications are relative.

Regarding complications following IO access, the rate of adverse events is described low in literature. Immediate complications include device failure, extravasation, fat embolism, fracture of the canullated bone and compartment syndrome of the concerning limb. The only prospective studies of IO access, including 553 adults, described none of these complications [21,25,26]. Late complications following IO access include osteomyelitis and bone marrow necrosis. Subsequent 4,270 IO cannulations of the sternum or tibia in children, infection rate was 0.6% [46]. Other authors found fewer or no infections at all. The risk for infection seems to correlate with exposure time of IO access exceeding several days, pre-existing bacteraemia, insufficient asepsis on insertion and usage as well as administration of hypertonic fluids [21,26,47]. Negative long-term effects for the cannulated bone, epiphysial plates or bone marrow in humans following IO infusion have not been demonstrated so far [48]. In our study population no IO related complication was detectable. Especially device-associated infection could be excluded by routine inspection of the insertion site three times daily and microbiological examination of each removed IO cannula.

Alternative vascular access techniques in the adult patient under resuscitation with impossible peripheral IV catheterization include CVC, ultrasound-guided catheterization of peripheral veins, and saphenous vein cutdown.

CVC provides vascular access for fluid resuscitation, vasoactive medication and antibiotics. Furthermore, it allows hemodynamic monitoring and cardiac pacing [12-15]. However, CVC is relatively time-consuming and associated with complications, especially in the emergency setting. Complication rates for CVC are reported around 15%, including malposition, arterial puncture, hematoma, pneumothorax, venous thrombosis and catheter related infections [12,14-17]. The average risk of CVC related infections in the critical ill patient accounts for 5.3 per 1,000 catheter days and leads to 80,000 CVC-associated bloodstream infections and consecutively up to 28,000 deaths in U.S. intensive care units each year. Mortality for hospitalized patients with CVC related infection accounts for 12-25%. The attributable cost per infection is estimated USD 34,508-56,000. Furthermore, CVC is relatively contraindicated for fibrinolytic therapy [10,49,50]. In our study none CVC related complications were detectable.

Ultrasound-guided peripheral IV access enables success rates of 92%. However, the necessary time adds up to 2.5-4 min for catheter cannulation itself, and up to 13 min for the whole procedure. Furthermore, this approach requires the ultrasound device and an experienced operator [4,5,51].

Saphenous vein cutdown is slow, less successful and associated with risks. The reported time need for this invasive vascular access procedure was 2-7.6 min with a success rate of 69-94%, when performed by experienced personnel. Trauma to the lower extremities might preclude saphenous vein cutdown. Time effect of administered drugs and fluids to the saphenous vein may be delayed due to long distance between vein cutdown and the heart, especially in shock conditions with impaired circulation [52-55].

Several potential limitations of this study have to be addressed. First, the sample size of this study is small due to its preliminary analysis of the first 10 consecutive patients. However, there was a statistically significant difference in procedure times of enabling vascular access, resulting in a time benefit for the IO approach (p < 0.001). Second, a potential bias of the investigators favouring towards the IO access was limited by performing both vascular access methods simultaneously by two independent operators. Third, differences in access success rates on first attempt and procedure times due to unequal experience in the applied techniques were limited by determining each participant to be an experienced consultant with long lasting expertise in resuscitation in the emergency department following standardized protocols.

As a consequence of the present study, we continued the IO vascular access protocol as a bridging procedure in adult patients under resuscitation in the emergency department with impossible peripheral IV access. To evaluate the handling of different IO access devices, we included different IO systems in our modified protocol of the ongoing trial.

## Conclusion

IO vascular access is a safe, reliable and rapid option in the acute setting of adult patients under resuscitation with inaccessible peripheral veins in the emergency department. Compared to CVC, IO cannulation is more successful on first attempt and requires significantly less time. However, IO access is not a surrogate for CVC and cannot replace it. Complications following IO access are rare, providing correct indication, training skills and adequate handling. Therefore, a change in practice from CVC to immediate IO access for the initial emergency resuscitation should be strongly considered as a reasonable bridging technique to increase patients' safety in the emergency department, if peripheral IV access was attempted unsuccessful three times for a maximum duration of 2 min. These findings are in accordance with current guidelines of the American Heart Association and the European Resuscitation Council [10,11]. Based on our findings, further prospective large-scale randomized trials are necessary to provide the treating physician with clear, evidence-based guidelines in the future.

## Abbreviations

CVC: Central venous catheterization; IO: Intraosseous; IV: Intravenous.

## Competing interests

The authors declare that they have no competing interests.

## Authors' contributions

BAL conceived the study, acted as primary physician conducting data acquisition, recruiting subjects, analyzed results and drafted the manuscript. CK and KGK assisted with initial study design, analysed results, helped with the statistic workup and helped draft the manuscript. VBo and JS assisted in testing the subjects, data acquisition and its analysis. WM assisted with study design and drafting of the manuscript. VBr assisted with study design, second observer for data acquisition, result analyzing, statistic workup and drafting of the manuscript. All authors read and approved the final manuscript.
